# Frataxin Is Localized to Both the Chloroplast and Mitochondrion and Is Involved in Chloroplast Fe-S Protein Function in Arabidopsis

**DOI:** 10.1371/journal.pone.0141443

**Published:** 2015-10-30

**Authors:** Valeria R. Turowski, Cindy Aknin, Maria V. Maliandi, Celeste Buchensky, Laura Leaden, Diego A. Peralta, Maria V. Busi, Alejandro Araya, Diego F. Gomez-Casati

**Affiliations:** 1 Centro de Estudios Fotosintéticos y Bioquímicos (CEFOBI-CONICET), Universidad Nacional de Rosario, Suipacha 531, 2000, Rosario, Argentina; 2 UMR5234 Microbiologie Fondamentale et Pathogénicité, Centre National de la Recherche Scientifique and Université Bordeaux-Segalen, 146 rue Léo Saignat, 33076, Bordeaux cedex, France; 3 Instituto de Investigaciones Biotecnológicas-Instituto Tecnológico de Chascomús (IIB-INTECH) CONICET/UNSAM, Camino de Circunvaación Km 6, 7130, Chascomús, Argentina; 4 Centre National de la Recherche Scientifique & UMR 1332 –Biologie du Fruit et Pathologie, Institute National de la Recherche Agronomique (INRA) Bordeaux Aquitaine, 71 avenue Edouard Bourlaux, 33882, Villenave D’Ornon, France; King's College London, UNITED KINGDOM

## Abstract

Frataxin plays a key role in eukaryotic cellular iron metabolism, particularly in mitochondrial heme and iron-sulfur (Fe-S) cluster biosynthesis. However, its precise role has yet to be elucidated. In this work, we studied the subcellular localization of Arabidopsis frataxin, AtFH, using confocal microscopy, and found a novel dual localization for this protein. We demonstrate that plant frataxin is targeted to both the mitochondria and the chloroplast, where it may play a role in Fe-S cluster metabolism as suggested by functional studies on nitrite reductase (NIR) and ferredoxin (Fd), two Fe-S containing chloroplast proteins, in AtFH deficient plants. Our results indicate that frataxin deficiency alters the normal functioning of chloroplasts by affecting the levels of Fe, chlorophyll, and the photosynthetic electron transport chain in this organelle.

## Introduction

Frataxin is a ubiquitous protein that is present in most organisms, from bacteria and fungi, mammals, and plants. In eukaryotes, frataxin has been described as a nuclear-encoded mitochondrial protein, but it is functional also in amitochondriate organisms [1, 2]. Frataxin deficiency is associated with the Friedreich’s ataxia (FRDA) phenotype, a cardio- and neuro-degenerative disease in humans [3, 4]. The structure of frataxin has been conserved throughout evolution, suggesting that it could have the same function in all organisms [5–7]. In general, all frataxin orthologs are able to bind iron, implicating them in such diverse physiological roles as: (i) iron homeostasis [4]; (ii) respiration and energy conversion [8]; (iii) regulator of Fe-S cluster formation [9]; (iv) biogenesis of Fe-S proteins [10–12]; (v) iron chaperone and storage [13, 14]; (vi) heme metabolism [15, 16] and (vii) REDOX control, ferroxidase activity and protection against oxidative damage associated with NO production [7, 17–21]. Thus, experimental evidence suggests that in eukaryotes, the frataxin protein plays a role in several processes associated with mitochondrial energy metabolism and Fe homeostasis.

Several studies have shown that a frataxin deficiency results in the over-accumulation of Fe in the mitochondrion and reduced activity in several Fe-S and heme proteins, associated with a decrease in ATP levels and impaired mitochondrial function [8, 10, 11, 22–24]. The association of frataxin with some proteins related to the Fe-S cluster biosynthetic machinery implies an important role for frataxin in this process [25].

Fe-S clusters are ubiquitous inorganic cofactors found in a large number of proteins involved in various physiological processes such as electron transfer, accumulation of Fe, Fe homeostasis, photosynthesis, catalysis, nucleic acid metabolism, and gene regulation [26](and references therein). The production of Fe-S groups is carried out by complex enzymatic machinery that incorporates iron and uses cysteine as a source of sulfur. The Fe-S groups are assembled while associated with scaffold proteins and are then inserted into specific apoproteins [27].

Three different types of Fe-S cluster biosynthetic systems have been described: (i) the *Nitrogen Fixation* (NIF) system required for the biogenesis of nitrogenase in azothropic bacteria [28, 29]; (ii) the *Iron-Sulfur Cluster* (ISC) system, a ubiquitous mechanism for the maturation of Fe-S proteins found in some bacteria and mitochondria [30]; and (iii) the *Sulfur Utilization Factor* (SUF) found in many bacteria, archaea, and plant chloroplasts. It has been proposed that this third system is closely related to the formation of Fe-S groups under conditions of oxidative stress and/or iron deficiency [26, 31–34]. In addition, a fourth “incomplete” system in eukaryotic cells, the *Cytosolic Iron-sulfur protein Assembly* system (CIA) was recently described. To date, little is known about the CIA system, but it is thought that it depends on some components from the mitochondria and the ISC system [27, 35]. All three Fe-S biosynthetic systems, excepting the CIA, have in common the participation of a cysteine desulfurase that provides the sulfur moiety from cysteine and a Fe-S scaffold protein for Fe-S cluster assembly.

A large number of Fe-S proteins have been identified in all plant cellular compartments. Moreover, several Arabidopsis genes have been characterized, revealing that the plastids, mitochondria and the cytosol have their own, albeit not entirely independent, Fe-S assembly machinery [35]. Based on the evolutionary origin of the genes associated with this function in different plant organelles,, it has been proposed that the chloroplast SUF machinery for the synthesis of Fe-S proteins is derived from cyanobacteria, while mitochondria use an ISC system that originated in the proteobacteria [35, 36].

In this sense, the genome of Arabidopsis encodes two isoforms of cysteine desulfurase, one belonging to the chloroplast SUF (AtNfs2), and the other to the mitochondrial ISC system (AtNfs1) [35]. In addition, the Arabidopsis genome contains genes for the scaffold proteins that participate in Fe-S synthesis in the mitochondria or chloroplasts; however Arabidopsis has only one gene for frataxin (*AtFH*) which plays a critical role in the biogenesis and maturation of Fe-S clusters and hemeproteins in mitochondria [16, 23, 37, 38]. Frataxin has been reported to interact with different proteins involved in the Fe-S biosynthetic pathway, such as Nfs1, modifying its catalytic activity [39, 40] [38]. In addition, functional interactions have also been reported between frataxin and Isu1, Isd11, and also with other mitochondrial proteins such as HSC20 and succinate dehydrogenase subunits [39, 41–44], showing the importance of frataxin in all stages of Fe-S cluster biogenesis performed by the ISC system. Our study shows for the first time clear evidence that frataxin is localized to both mitochondria and chloroplasts, and highlights its physiological relevance to chloroplast functions in Arabidopsis.

## Materials and Methods

### Plant material and growth conditions

*Arabidopsis thaliana* ecotype Columbia (Col-0) was used as the wild-type line. Two independent transgenic lines expressing the AtFH fragment in antisense orientation under the control of the cauliflower mosaic virus 35S (CaMV35S) promoter were used as frataxin deficient lines, *as-AtFH-1* and *as-AtFH-2* [16]. The transgenic line *CaMV35S-u-ATP9*, expressing an unedited *ATP9* gene, and showing impaired mitochondrial function, was used as control of NIR activity measurements [45]. Transgenic AtFH-GFP plants were constructed by transformation with the pZP212 vector [46] containing the coding sequence of the AtFH gene (564 bp) fused to the enhanced green fluorescence protein (GFP) ORF [47]. Transgenic *as-AtFH* lines and AtFH-GFP plants were selected on MS agar medium containing either 20 μg/ml hygromycin or 50 μg/ml kanamycin, respectively. After 14 days, plants were transferred to soil and grown in a greenhouse at 25°C under fluorescent lamps (Grolux, Sylvania, Danvers, MA, USA and Cool White, Philips, Amsterdam, The Netherlands) with an intensity of 150 μmol^.^m^-2.^s^-1^ using a 16 h light ⁄ 8 h dark photoperiod.

### Production of AtFH-GFP protoplasts and confocal microscopy

Protoplasts were prepared from young leaves of 3-week-old Arabidopsis plants essentially as described [48]. Leaves were cut into 1 mm strips with sharp razor blades and placed in 24-well plates containing 500 μl of 1.5% cellulase (Sigma) and 0.4% driselase (Sigma) in 20 mM MES buffer, pH 5.7, containing 0.4 M mannitol, 20 mM KCl, 10 mM CaCl_2_ and 1% w/v BSA. Cell wall digestion was performed at 25°C for approximately 3 h with constant agitation (60 rpm), and the protoplasts were collected by centrifugation and washed in the same buffer without enzymes. Protoplasts were treated with 0.1 μM MitoTracker Orange (Invitrogen) for 15 min and washed three times with MS medium. Protoplasts were embedded by mixing 1 volume of 1% low melting Sea Plaque GTG agarose (FMC BioProducts) with 1 volume of protoplast suspension. A drop of this mixture was placed on microscope slide and overlaid with a coverslip. Images were acquired with a Leica TCS SP5 confocal microscope (Leica Microsystems, Wetzlar, Germany). The transmission micrographs of non-fluorescent protoplast structures were acquired using the manufacturer’s filter settings. GFP fluorescence was excited with an argon laser (488 nm) and detected at 510 nm. Mitochondria were identified by the fluorescence of MitoTracker excited at 543 nm with a green helium neon laser and detected at 576 nm. Chlorophyll auto fluorescence (red) was detected at 650 nm when excited at 488 nm with the argon laser.

### Isolation of chloroplasts and mitochondria

Chloroplasts were purified as previously described with some modifications [49]. About 100 g of leaves from 5- to 6-week old *Arabidopsis thaliana* plants (*as-AtFH-1* and *-2*, and *u-ATP9* lines) were excised and homogenized in 50 ml of cold extraction buffer (50 mM HEPES, pH 8; 330 mM Sorbitol, 2 mM EDTA, 1 mM MgCl_2_, 5 mM ascorbic acid, 0.05% (w/v) BSA, 2 mM PMSF) using an Omni Mixer (Omni International, Kennesaw, GA, USA). The homogenate was then clarified by filtration and centrifuged for 5 min at 2,500 *x* g at 4°C. The pellet was gently suspended in 3 ml of HEPES/sorbitol buffer (50 mM HEPES, pH 8, 330 mM sorbitol), layered onto a base of 3 ml of HEPES/Sorbitol buffer containing 35% w/v Percoll, and centrifuged at 2,500 *x* g for 10 min. at 4°C. This procedure was repeated four times. The chloroplast suspension was frozen in liquid nitrogen and stored at -80°C until use. Highly purified mitochondria from *A*. *thaliana* flowers were isolated as previously described [23]. Under these conditions, the mitochondrial fraction is devoid of cytoplasmic and plastid contamination. Mitochondria were recovered in a buffer containing 300 mM mannitol and 10 mM K_2_HPO_4_ (pH 7.4).

### Chlorophyll fluorescence parameters

Chlorophyll fluorescence was measured using conditions adapted from Maxwell and Johnson [50]. Seedlings were germinated on MS medium either with hygromycin at 20 μg/ml (for *as-AtFH-1 and -2* plants) or without antibiotics (wt plants), and then transferred to soil. After seven weeks chlorophyll fluorescence was measured *in vivo* using a fluorometer (OS-500 pulse amplitude modulated, Qubit Systems). Leaves were dark-adapted for 15 min and the measuring beam was then turned on and minimal fluorescence (F0) was measured. Further, leaves were exposed to a saturating flash (3,000 μmol m^–2^ s^–1^) to determine maximal fluorescence (Fm). An actinic light to drive photosynthesis (100 μmol m^–2^ s^–1^) was then applied. After about 3 min., another saturating light flash allowed the maximum fluorescence in the light (F´m) to be measured. The level of fluorescence immediately before the saturating flash is termed Ft. Once instantaneous fluorescence had returned to the level of F0, the (F´0) was calculated.

The Quantum yield of PSII was calculated as ϕPSII = (F´m–F´t)/F´m; the Maximum Quantum yield of PSII was calculated as Fv/Fm = (Fm–F0) / Fm; the Photochemical Quenching was calculated as qP = (F´m-Ft) / (F´m-F´0) and the Non Photochemical Quenching was calculated as NPQ = (Fm-F’m) / F’m.

### Quantitative RT-PCR assay

Total RNA was isolated from leaves using the TRIzol reagent (Invitrogen). RNA integrity was checked on a 1% (w/v) agarose gel, and the concentration was determined by absorbance at 260 nm. cDNA was synthesized using random hexamer primers. The conditions used were those described in the Access RT-PCR system first strand protocol (Promega). Quantitative real time PCR was performed as previously described using a MiniOpticon2 apparatus (Bio-Rad) [51]. Amplification was initiated with a 2-min denaturation step at 94°C. The protocol consisted of 40 cycles at 96°C for 10 s, 60°C for 15 s, and 72°C for 1 min, followed by 10 min extension at 72°C. Fluorescence detection was performed at the end of each annealing step for 1 s. Melting curves for each PCR were determined by measuring the decrease in fluorescence with increasing temperature (from 65–98°C). The *actin2* gene (At3g18780) transcript was used as an internal reference, and relative quantification was performed using the 2^-∆∆Ct^ method [52].

### Protein extraction and western-blot analysis

The chloroplast or mitochondrial suspensions were disrupted by sonication (25% amplitude for 1 min) and centrifuged for 10 min at 7,000 x *g* at 4°C. The protein content of the supernatant was measured according to Bradford [53] using bovine serum albumin as protein standard. Ten to thirty micrograms of the protein extract was layered onto a 15% SDS polyacrylamide gel and electrophoresed for 1 h at 35 mA. Ferredoxin (Fd) was detected using a polyclonal anti-pea Fd antibody kindly provided by Dr. Nestor Carrillo (National University of Rosario), NAD9 was detected using a polyclonal antibody against the wheat NAD9 subunit kindly provided by Dr. Daniel Gonzalez (National University of Litoral), and ADPGlc PPase was detected using anti-ADPGlc PPase from spinach leaf [54]. AtFH was detected using affinity-purified antibodies raised against recombinant AtFH [55]. The relative abundance of individual protein bands was quantified using the Gel Pro Analyzer program 4.0 (Media Cybernetics, Silver Spring, MD, USA).

### Additional methods

The chlorophyll content of isolated chloroplasts was quantified according to the method of Arnon [56]. Chloroplastic iron was quantified by the ferrozine method following an initial treatment with acid to release complexed iron [19, 57]. NIR activity was determined in isolated chloroplasts based on the method described by Takahashi et al. [58]. AtFH-Fe or BSA-Fe were obtained by preincubation of 3 μM solutions of AtFH or BSA with 30 μM Fe_2_SO_4_ for 10 min at 30°C in 50 mM potassium phosphate, 1 mM DTT, pH 7.5. All determinations were performed at least in triplicate, and the average values ± SD are reported. The significance of differences was determined using Student’s t-test. Statistically different values (*P* < 0.05) are shown with an asterisk in the tables and figures.

## Results

### AtFH localizes to the chloroplasts and mitochondria

In order to predict the subcellular location of Arabidopsis frataxin (AtFH), *in silico* analyses were performed (Table 1). A higher probability that frataxin is targeted to mitochondria was found using programs based on different algorithms [59–62]. Moreover, it was possible to predict that AtFH could be imported into chloroplasts with a significant score as well. In addition, predictions using the ATP2 program, which specifically evaluates the probability of dual targeting, agreed that AtFH is likely localized to both the mitochondria and chloroplasts (Table 1).

**Table 1:** Predicted subcellular localization of AtFH (At4g03240).

PREDICTOR	LOCALIZATION	SCORE
TargetP^a^	Mitochondria	0.858
	Chloroplast	0.177
MitoProt^b^	Mitochondria	0.998
ChloroP^c^	Chloroplast	0.507
GTP_ pp^d^	Mitochondria	0.695
	Chloroplast	0.587
GTP_ref^d^	Chloroplast	0.683
	Mitochondria	0.548
ATP2^d^	Dual	0.759

^a^ TargetP 1.1 Server (http://www.cbs.dtu.dk/services/TargetP)

^b^ MitoProt II–v1.101 (http://ihg.gsf.de/ihg/mitoprot.html)

^c^ ChloroP 1.1 Server (http://www.cbs.dtu.dk/services/ChloroP/)

^d^ GTP—Green Targeting Predictor & ATP2—Ambiguous Targeting Predictor 2 (http://www.plantco.de/gtp).

**Fig 1:**
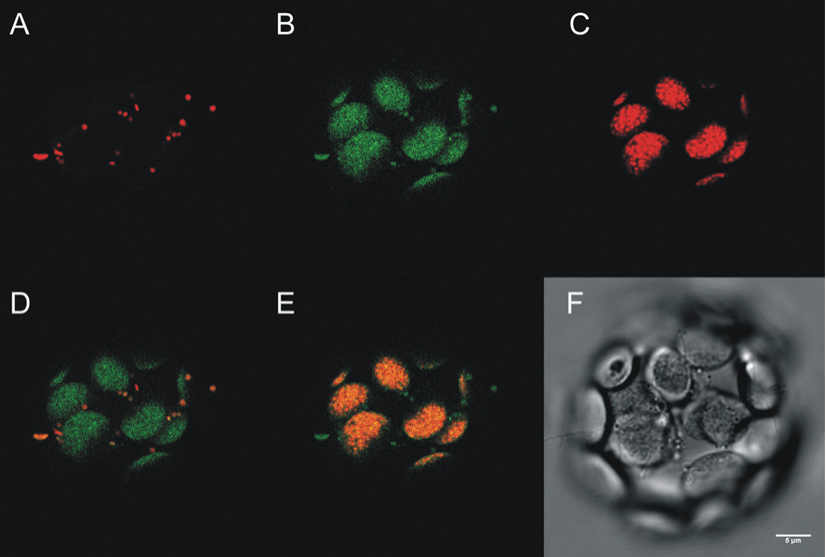
Subcellular localization of AtFH. The plasmid pZP212 containing the coding sequence of AtFH-GFP was introduced into Arabidopsis thaliana Col-0 plants by the floral dip method. Protoplasts were isolated from the resulting transgenic plants and analyzed by confocal microscopy. (A) Mitotracker staining showing mitochondria (excitation 543 nm/emission 576 nm); (B) GFP fluorescence (excitation 488 nm/emission 510 nm); (C) Chlorophyll autofluorescence (excitation 488 nm/emission 650 nm); (D) Overlay of A and B showing coincidence of GFP localization and Mitotracker (yellow); (E) Overlay of B and C showing the coincidence of GFP localization and chlorophyll autofluorescence (yellow); (F) Phase contrast image of the protoplast analyzed.

**Fig 2:**
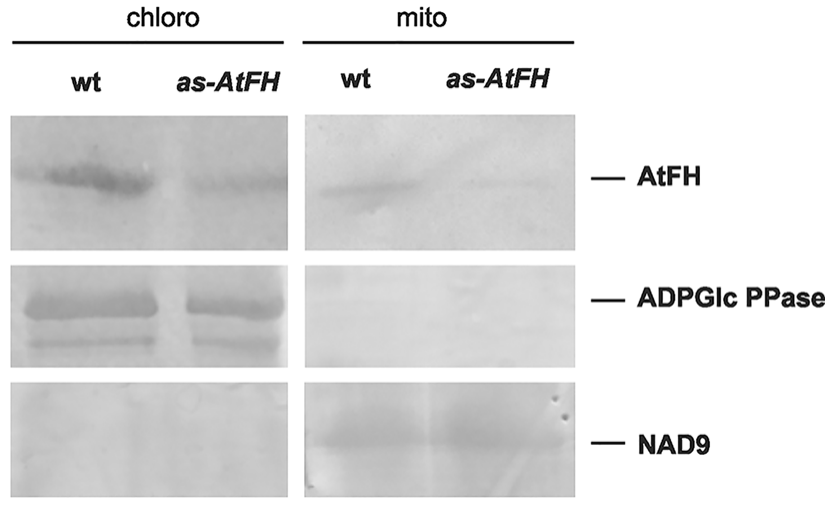
Analysis of the presence of AtFH in isolated chloroplasts and mitochondria from *A*. *thaliana* by western blot in wt and *as-AtFH-1* plants. AtFH was detected using anti-AtFH antibodies. Anti-ADPGlc PPase and anti-NAD9 antibodies were used as controls to asses the purity of chloroplasts and mitochondria, respectively.

In order to test these predictions, we constructed *Arabidopsis thaliana* transgenic plants carrying the coding sequence of the AtFH gene fused to the green fluorescent protein (AtFH-GFP; see Materials and Methods) under control of the constitutive CaMV 35S promoter. Following plant transformation, GFP fluorescence was examined in leaf protoplasts via confocal microscopy. AtFH-GFP was found to be associated with mitochondria as indicated by the overlapping of GFP fluorescence with the Mitotracker staining (Fig 1A, 1B and 1D). Interestingly, a significant accumulation of AtFH-GFP was detected in the chloroplast compartment as observed by the overlapping of the green GFP fluorescence with chlorophyll auto fluorescence (Fig 1B, 1C and 1E). In order to evaluate the localization of AtFH, the presence of the protein in chloroplasts and mitochondria was also confirmed by immunoblotting using immunopurified anti-AtFH antibodies. The absence of the NAD9 mitochondrial protein in chloroplastic preparations, and the absence of chloroplastic ADPGlc PPase subunits in mitochondria, were used as controls to asses the purity of chloroplasts and mitochondria, respectively (Fig 2).

### Physiological effects of altered AtFH expression in chloroplasts

**Fig 3:**
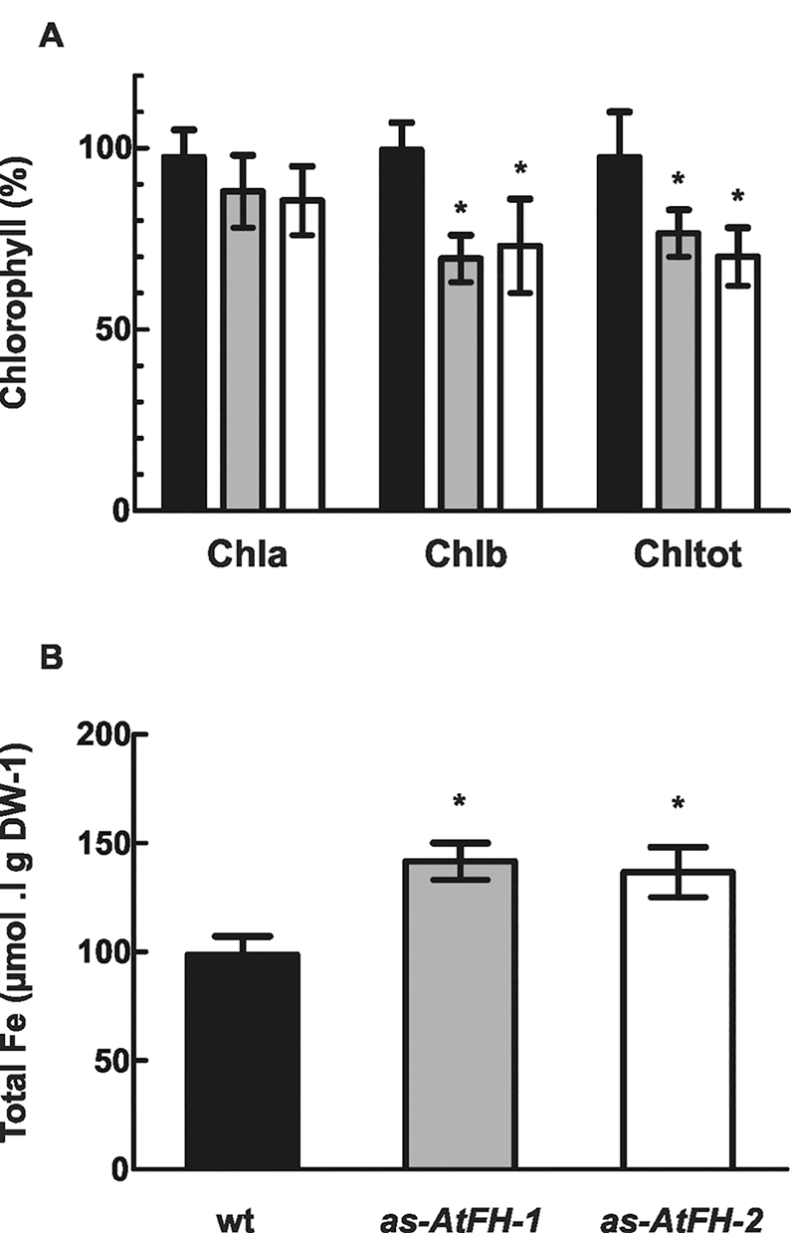
Quantification of chlorophyll and iron contents. (A) Quantification of chlorophyll a, b and total chlorophyll in wt (black bars), *as-AtFH-1* (grey bars), and *as-AtFH-2* (white bars) plants. (B) Analysis of total iron content in wt and *as-AtFH* plants. Asterisks indicate a statistically different result from the control value (P < 0.05). Values are the mean ± standard deviation of at least four independent replicates.

Previously, we established that frataxin deficiency results in severe changes in mitochondrial function in *Arabidopsis* [23] as previously shown in other eukaryotes [8, 11, 12]. To determine whether frataxin has a role in chloroplast functions as suggested by the ability of the AtFH N-terminal signal sequence to target chloroplasts, we quantified the chlorophyll and total Fe content of isolated chloroplasts from frataxin deficient *as-AtFH-1* and *as-AtFH-2* plants. The total chlorophyll content decreased between 20 and 30%, with a significant decrease of ~30% in chlorophyll b (Fig 3A). In contrast, the total Fe content increased ~40% in *as-AtFH* chloroplasts with respect to the wt controls (Fig 3B). Since these results suggest a possible alteration of chloroplast function, we decided to investigate the photosynthetic capacity in frataxin deficient plants. For this purpose, chlorophyll fluorescence parameters were compared in leaves of *as-AtFH* and wt plants using light conditions adapted from Maxwell et al. (2000) [50]. The Fv/Fm ratios, an indicator of the plant photosynthetic performance (intactness), were similar; however, small but significant differences were found for the Quantum yield of PSII (ϕ PSII), which indirectly estimates the flux of electrons out of PSII, and the Photochemical quenching (qP), which reflects the ratio of open PSII reaction centers after electron transfer to PSI (Table 2). Both parameters show a slight reduction of ~10–13% when comparing frataxin-deficient to wt control leaves. In addition, an important decrease of ~39–43% in the non-photochemical quenching (NPQ), which is related to the dissipation of excess excitation energy as heat, was found in *as-AtFH* plants (Table 2). Taken together, these data indicate that frataxin deficiency alters the normal functioning of chloroplasts by affecting the levels of Fe, chlorophyll, and the photosynthetic electron transport chain in this organelle.

**Table 2:** Photosynthetic parameters in *as-AtFH* and wt leaves.

line	Fv/Fm	ϕ PSII	qP	NPQ
wt	0.795 ± 0.035	0.688 ± 0.036	0.809 ± 0.034	0.316 ± 0.048
*as-AtFH-1*	0.789 ± 0.029	0.617 ± 0.029*	0.729 ± 0.044*	0.194 ± 0.058*
*as-AtFH-2*	0.775 ± 0.042	0.597 ± 0.024*	0.713 ± 0.027*	0.183 ± 0.043*

Fv/Fm, Maximum quantum yield of PSII; ϕ PSII, Quantum yield of PSII; qP, Photochemical quenching; NPQ, Non-photochemical quenching. Values are the mean ± standard deviation of at least three leaves from 10 individual plants.

Asterisks (*) indicate statistically different results (P < 0.05).

### AtFH deficiency affects ferredoxin levels and nitrite reductase activity in chloroplasts

**Fig 4:**
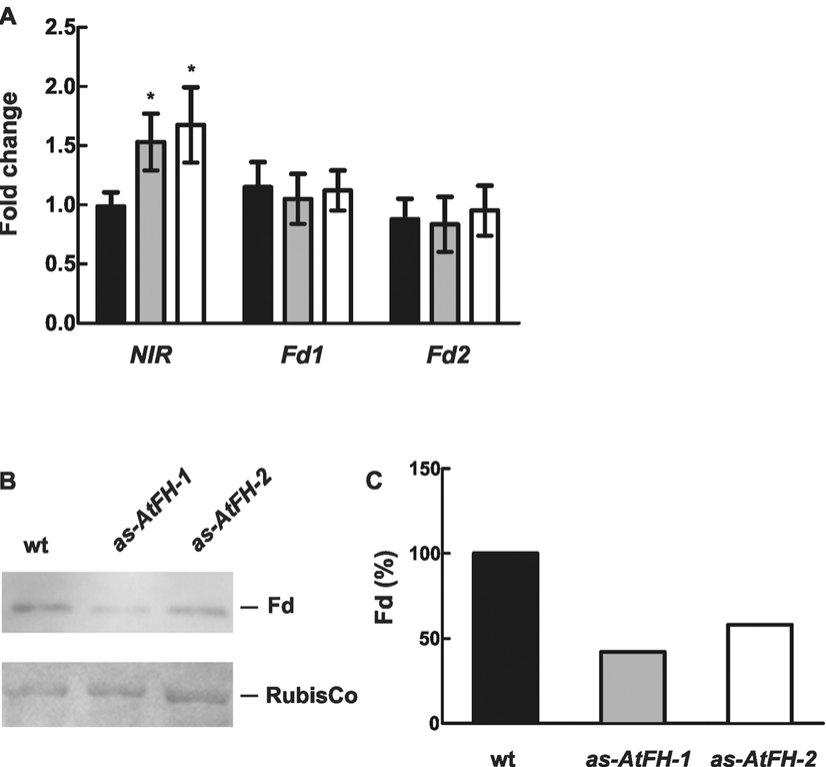
Analysis of chloroplastic Fe-S genes and proteins. (A) qRT-PCR analysis of genes encoding chloroplastic Fe-S proteins. NIR: nitrite reductase (AT2g15620.1), Fd1: ferredoxin 1 (At1g10960), Fd2: ferredoxin 2 (At1g60950). RNA was extracted from leaves of wt (black bars), *as-AtFH-1* (grey bars) and *as-AtFH-2* (white bars) plants. Asterisks indicate a statistically different result from the control value (P < 0.05). Bars represent mean values (error bars ± standard deviation) of three independent experiments. The relative expression levels of the transcripts are shown as fold-changes with respect to *actin2* mRNA levels. (B) Western blot analysis of total Fd levels in chloroplasts of wt and *as-AtFH* plants using a specific anti-Fd IgG. Ponceau staining of Rubisco large subunit is shown as a loading control. (C) Quantification of Fd signals from B in wt and *as-AtFH* plants.

**Fig 5:**
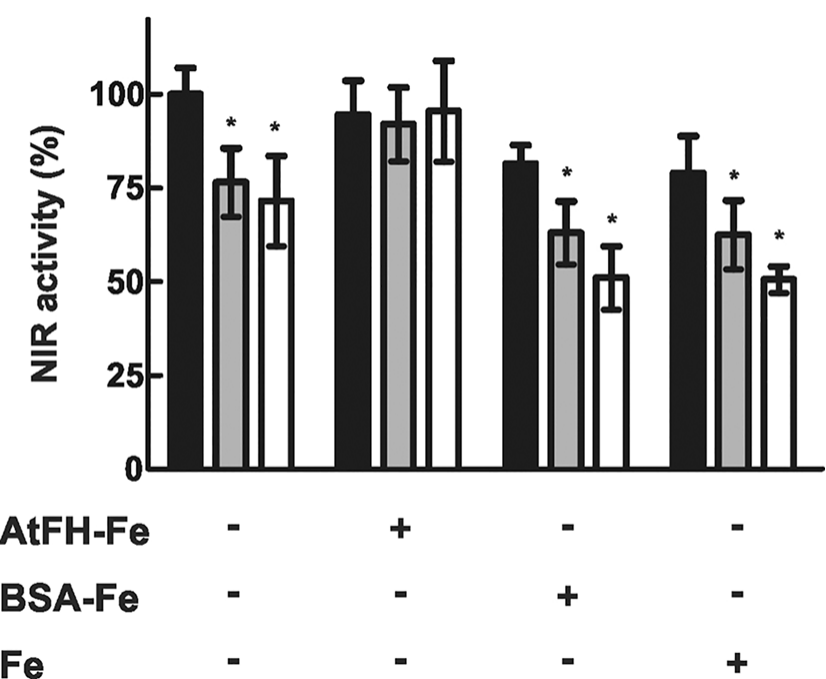
Determination of NIR activity. NIR activity was assayed in extracts of isolated chloroplasts with no additions (control) or in the presence of AtFH-Fe, BSA-Fe or Fe alone, in wt (black bars), *as-AtFH-1* (grey bars) and *as-AtFH-2* (white bars) plants. The asterisk indicates values statistically different from the control (P < 0.05). Columns represent mean values (error bars ± standard deviation) of at least three independent experiments.

Several independent reports have linked frataxin to iron sulfur cluster assembly [10, 23, 63–65]. Moreover, we found that the activity of some mitochondrial Fe-S proteins was affected in frataxin deficient plants [23]. To investigate whether a similar effect occurs in chloroplasts, we determined the mRNA levels for the genes encoding the chloroplast Fe–S proteins nitrite reductase (NIR) and ferredoxin (Fd) in *as-AtFH* and wt plants using qRT-PCR. While NIR mRNA levels showed an increase of ~1.5 to 1.8-fold in *as-AtFH* lines compared to wt plants, no significant differences were observed for *Fd* mRNA (Fig 4A). Furthermore, to determine whether the Fd and NIR transcript levels observed in *as-AtFH* plants have an influence on protein accumulation or function, we analyzed (i) ferredoxin levels by western blot analysis using a polyclonal antibody raised against pea Fd, and (ii) the activity of NIR, which is an Fe-S enzyme involved in the second step of nitrate assimilation in plants (see Materials and Methods). The results showed a reduction in Fd levels of ~55% and 40% in *as-AtFH-1* and *as-AtFH-2* plants, respectively, compared to wt plants (Fig 4B and 4C). A similar result was found for NIR showing a 25–30% decrease in enzyme activity in *as-AtFH* lines compared to wt plants (see Fig 5). Taken together, these results suggest that the decrease in Fd levels and NIR activity occurs mainly at the posttranslational level, possibly because AtFH may be required for both the normal turnover and/or functionality of these chloroplast Fe-S proteins.

### Frataxin is required for full NIR activity in chloroplasts

One explanation for the effect of frataxin deficiency on NIR activity could be the alteration in the Fe-S cofactor. Therefore, we analyzed NIR activity from the wt and both *as-AtFH* lines after incubating chloroplast extracts with the recombinant AtFH pre-equilibrated with Fe^2+^ [55]. Chloroplast extracts incubated with BSA-Fe or Fe alone were used as the control (Fig 5). The NIR activity was fully recovered after incubating the extracts with AtFH-Fe. By contrast, NIR activity was not recovered, and a decrease in activity was observed in wt and *as-AtFH* lines when BSA-Fe, or Fe alone, was added to chloroplast extracts. This inhibitory effect is probably due to the production of free radicals by iron when it is not complexed with frataxin. In addition, the isolated chloroplasts of *u-ATP9* plants having impaired mitochondrial function [48, 54] showed similar levels of NIR catalytic activity compared to wt chloroplasts, suggesting that the decrease of NIR activity observed in the *as-AtFH* plants is specific (see S1 Fig).

## Discussion

Consistent with the mitochondrial localization of frataxin, changes in expression and/or deficiency of this protein are associated with mitochondrial dysfunction and iron metabolism disorders [1, 4]. In humans and yeast, the N-terminal region of the protein directs its import into mitochondria, followed by two proteolytic cleavages carried out by a mitochondrial processing peptidase (MPP) [66, 67]. However, frataxin is not exclusively located in this organelle, since the presence of a functional extra-mitochondrial frataxin pool able to interact and modulate the activity of cytosolic aconitase/iron regulatory protein-1 (IRP1) has been reported [68]. Moreover, frataxin interacts with IscU1, a cytoplasmic isoform of the Fe-S cluster biosynthetic machinery, suggesting a role for frataxin in the biogenesis of Fe-S clusters outside of the mitochondrion [25].

In Arabidopsis, we have previously described the crucial role of frataxin in mitochondria, where it is able to modulate the activity of Fe–S proteins, take part in stress responses, and participate in heme synthesis [16, 23, 37]. Despite these advances, the function of this protein in plants has not been completely elucidated [27], possibly because AtFH could be functioning in other cellular compartments. Defining the subcellular localization of a protein is the starting point towards understanding its metabolic function [69, 70]. Lan et al [71] have reported the presence of AtFH in the Arabidopsis root proteome; however, there is no evidence for the presence of frataxin in plant organelles from proteomic data. Other proteins involved in Fe-S cluster biosynthesis in mitochondria (such as AtHscB, AtSufE1, and AtIsu2 and 3) or chloroplasts (such as AtSufE2 and CplscA) were not found in any available proteome, probably due to low expression levels, and their presence in organelles was demonstrated by experiments using GFP fusions [72–76].

This study is the first to examine the cellular location of Arabidopsis frataxin using *in vivo* methods, and we found that the protein is targeted to both mitochondria and chloroplasts (Figs 1 and 2). Although the mitochondrial targeting of AtFH was expected from the phylogenetic distribution of frataxin [1, 77], its localization to the chloroplast had not been previously reported. The dual localization of proteins into energetic organelles in plants that fulfill analogous functions, such as mitochondria and chloroplasts, is not unusual [78, 79]. More than 100 proteins have been described that are localized in mitochondria and chloroplasts and are involved in crucial cellular functions such as protein synthesis, DNA and RNA metabolism, protein fate, stress, detoxification, and energy metabolism [78, 80]. We present here evidence that AtFH is targeted to both mitochondria and chloroplasts, consistent with its putative role as iron donor for Fe-S cluster formation, which occurs in both compartments.

The presence of frataxin homologues in proteobacterial genomes, despite the absence of orthologs in gram-positive bacteria, led to the inference that this protein is targeted to the mitochondrion in eukaryotes due to the origin of this organelle. Moreover, the co-occurrence of frataxin together with other proteins related to Fe-S cluster assembly suggests that it has a role as an iron donor in the eukaryotic mitochondrial ISC system [1, 77]. Thus, the unexpected finding of a structural frataxin homolog in Gram positive bacteria was surprising [81]. The frataxin homologue from *Bacillus subtilis* participates in the biosynthesis of heme and Fe-S groups, although in the latter it is associated with the SUF system [81, 82]. In this sense it is possible to suggest that, regardless of its phylogenetic origin, frataxin is an iron donor acting on the same metabolic pathways. Therefore, our results indicate that, even with an evolutionary history of frataxin that is linked to the mitochondrion, AtFH is localized to both mitochondria and chloroplasts, where it has similar functions.

Chloroplasts possess SUF-type machinery for the synthesis of their own Fe-S proteins, and this is different than the mitochondrial ISC system [35, 36]. In chloroplasts, Fe-S clusters play a key role in photosynthetic electron transport as well as in nitrogen and sulfur assimilation [83]. Thus, we hypothesized that AtFH could be involved in the metabolism of Fe-S clusters in chloroplasts and/or participate in the coordination of the SUF and ISC systems. If this is indeed the case, AtFH would play a role as a modulator of the two types of Fe-S cluster biosynthesis systems, regulating the activity of proteins involved in the pathways. Previously, we found that frataxin deficiency affects the expression of some genes involved in tetrapyrrole synthesis [16]. Since tetrapyrroles participate in the common pathway for chlorophyll and heme synthesis, it is possible that the observed reduction in photosynthetic pigments (Fig 3A) is due to changes in the synthesis of tetrapyrrole precursor metabolites. Alternatively, AtFH deficiency may affect the activity of chlorophyll *a* oxygenase, a Rieske 2Fe-2S cluster-containing enzyme that catalyzes the interconversion of chlorophyll *a* to *b* [84].

To investigate these possibilities, we first analyzed the chlorophyll and iron contents in chloroplasts of the *as-AtFH* lines and wt plants. As previously reported, frataxin deficiency is correlated with an increase in mitochondrial iron content [4, 19]. An analogous result was found for total Fe content in frataxin-deficient *Arabidopsis* plants (Fig 3B). The higher iron content in chloroplasts of *as-AtFH* lines highlights the role of frataxin in maintaining the homeostasis of this metal ion in the cell. Moreover, AtFH deficient plants showed altered photosynthetic capacity which was not related to variations in the composition of the PSII reaction centers, since no differences in the Fm/Fv parameter were observed between *as-AtFH* and wt plants. An AtFH deficiency would shift the redox state of the electron acceptors from the PSII with a consequent decrease in the rate of electron transport. Hence, impaired re-oxidation of the plastoquinone pool leads to a lower efficiency of photosystem II. Similar findings were obtained from Arabidopsis plants lacking the chloroplastic proteins Nfu2 or AtNfs2 in which minor values of ФPSII and qP correlated with lower amounts of iron-sulfur proteins associated with PSI [85, 86]. On the other hand, it is well established that chlorophyll b is the most abundant pigment in the light harvesting complex II (LHCII) that is involved in the dissipation of excess light energy as heat [87, 88]. The lower levels of non-photochemical quenching (energy dissipated as heat) in *as-AtFH* plants could be due to the reduced amount of chlorophyll b (Fig 3A and Table 2).

It has been reported that frataxin deficiency causes a decrease in the activities of several mitochondrial Fe-S proteins [11, 23, 64, 65]. If frataxin plays a similar role in chloroplasts, then an analogous behavior should be observed in some plastid Fe-S proteins. This is the case for NIR, a chloroplast Fe-S containing protein. Interestingly, while an increase in NIR mRNA levels was detected, the NIR catalytic activity was reduced in frataxin deficient plants (Figs 4 and 5); however, no reduction of NIR activity was observed in *u-ATP9* line showing dysfunctional mitochondria. These results suggest that frataxin deficiency may be related to the Fe–S cluster assembly in NIR. In the case of ferredoxin (Fd), the major iron-containing protein in photosynthetic organisms that is central to reductive metabolism in the chloroplast, a 40–55% reduction in protein abundance was found in AtFH deficient plants. It is assumed that the reduction in Fd may occur by protein degradation related to an inability to form the Fd holo-form. Analogous observations have been reported for plants deficient in chloroplastic cysteine desulfurase, which is responsible for sulfur delivery for Fe-S cluster synthesis [86].

An important result from our study is the full recovery of NIR activity observed after addition of iron-complexed AtFH to chloroplast extracts. This response may be interpreted as an effect of AtFH on the Fe-S cluster assembly to form the active NIR holo-protein. Similar results were previously described for aconitase, where human frataxin participates in the conversion of the inactive 3Fe-4S enzyme to the active 4Fe-4S form [14]. These results fully support the notion that frataxin is involved in Fe-S cluster biogenesis and promotes enzyme reactivation. Interestingly, control experiments show that the addition of Fe alone or combined with BSA results in a reduction of NIR activity that can be assumed to result from the production of free radicals by iron. The fact that inhibition was prevented in the presence of AtFH is a strong argument supporting the protective role of frataxin and its participation as an iron chaperone. Taken together, these results suggest that plant frataxin, in addition to restoring NIR activity, also plays an important role in protecting chloroplasts against oxidative damage.

In summary, the results presented here imply a novel role for frataxin in plant chloroplasts. We demonstrate that AtFH is targeted to both the mitochondria and chloroplasts, where it may play a role in maintaining the correct structure of Fe-S clusters and/or in maintaining their proper redox state to ensure the correct functioning of chloroplast iron sulfur proteins.

## Supporting Information

S1 Fig Determination of NIR activity. Determination of NIR activity in extracts of isolated chloroplasts with no additions (control) or in the presence of AtFH-Fe, BSA-Fe or Fe alone, in wt (white bars) or *u-ATP9* plants (black bars). Columns represent mean values (error bars ± SD) of at least three independent experiments.
(TIF)

## Abbreviations

AtFH: Arabidopsis frataxin homolog
NIR: nitrite reductase
Fd: ferredoxin
